# Implications of the new EU legislation on chemicals for Poisons Centres

**DOI:** 10.3109/15563650.2011.623129

**Published:** 2011-11-14

**Authors:** Ronald De Groot, Pieter Brekelmans, Jan Meulenbelt

**Affiliations:** 1 National Poisons Information Center, University Medical Center Utrecht, The Netherlands; 2 Division of Anesthesiology, Intensive Care and Emergency Medicine, University Medical Center Utrecht, The Netherlands; 3 Institute for Risk Assessment Sciences, Utrecht University, Utrecht, The Netherlands

**Keywords:** Poisons Centres practise, REACH Regulation, CLP Regulation, Cosmetic Products Regulation, Product notification

## Introduction

In the last 5 years, the legislation on chemicals was rigorously updated in the European Union (EU). The CLP (Classification, Labelling and Packaging of substances and mixtures) Regulation^1^ and REACH (Registration, Evaluation, Authorisation and Restriction of Chemicals) Regulation^2^ completely renew the rules on (risk) management of substances and mixtures. They will gradually replace a large variety of directives on chemicals, including the Dangerous Substances Directive, Dangerous Preparations Directive and Safety Data Sheet Directive. Furthermore, the new Cosmetic Products Regulation^3^, Plant Protection Products Regulation^4^ and forthcoming Biocidal Products Regulation^5^ replace the former directives on these product groups. See for more details and specific legislation numbers Table 1. As these new EU legislations are ‘regulations', they directly apply in the same way to every EU Member State after coming into force. Regulations do not allow Member States the freedom to interpret the ruling in different ways. The former ‘directives’ allowed EU Member States to adapt and/or complement the EU legislation with specific national requirements into National legislation.

**Table 1:** New legislation on chemicals in the European Union.

Old Directives	New Regulations		Relevance for Poisons Centres
Dangerous Substances Directive 67/548/EEC	Regulation on classification, labelling and packaging of substances and mixtures (CLP) Regulation (EC) No 1272/2008^1^	New hazard classification of substances. New hazard classification of mixtures.	
Dangerous Preparations Directive 1999/45/EC		Article 45	Harmonisation of product notification (requirements and data exchange format).
Safety Data Sheet Directive 91/155/EEC >60 Substances Directives	Registration, Evaluation, Authorisation and Restriction of Chemicals (REACH) Regulation (EC) No 1907/2006^2^	Annex II	Improvement of toxicological information in Safety Data Sheets.
Cosmetics Products Directive 76/768/ EEC	Cosmetic Products Regulation (EC) No 1223/2009^3^	Article 13	Notification of cosmetic products to a central EC database (Cosmetic Products Notification Portal). This information will be available for Poisons Centres and governmental authorities.
Plant Protection Products Directive 91/414/EEC	Plant Protection Products Regulation (EC) No 1107/2009^4^		The Plant Protection Products Regulation does not contain an article on product notification. Article 45 of the CLP Regulation applies also for this product group.
Biocidal Products Directive 98/8/EEC	Biocidal Products Regulation (EC) No XXXX/2011^5^ (forthcoming in 2011)	Article 63	For the notification of biocidal product information article 63 refers to article 45 of the CLP Regulation.

For Poisons Centres (PCs), the changes in legislation on chemicals predominantly affect the notification of product information by companies. The European Association of Poisons Centres and Clinical Toxicologists (EAPCCT), recognised as an important stakeholder, is actively involved in the discussions on the implementation of these legislative developments. The EAPCCT participates in projects of the European Commission (EC) on the notification of hazardous mixtures and of cosmetic products. This article describes the implications of the new EU legislation on chemicals for Poisons Centres.

## CLP Regulation

The CLP Regulation has entered into force on January 20, 2009. It will align the EU legislation on classification and labelling of substances and mixtures (for supply and use) to the United Nations Globally Harmonised System of Classification and Labelling of Chemicals (UN-GHS).^6^ UN-GHS provides harmonised classification criteria and hazard communication elements, aiming at global harmonisation of hazard classification. Poisons Centres will be confronted with a new hazard classification according to the CLP Regulation, introducing new health hazard classes and categories, with associated new hazard pictograms, signal words, Hazard(H)-statements and Precautionary(P)-statements as hazard communication elements.^7^ Especially relevant for PCs are the new P-statements with the phrase: ‘Call a POISON CENTER or doctor/physician'. In countries where the PCs are only open to inquiries from the health service and not from the general public (like in the UK and in the Netherlands) such statements on a product label could increase the inquiries from the general public and so have consequences for the poisons information supply in these countries.^7^ From December 2010, the new hazard classification is already obligatory for substances. For mixtures (replacing the old term ‘preparations'), the old and new classification will exist simultaneously until June 2015. Furthermore, the CLP Regulation will affect the notification of product (mixture) information by companies.

### Notification of product information on hazardous mixtures

The main task of Poisons Centres is to inform medical personnel and/or the public about symptoms and treatment of acute intoxications. To perform this task adequately, PCs need detailed information on the composition of products to which consumers can be exposed. In the CLP Regulation, paragraphs 1-3 of article 45 describe the notification of information on hazardous mixtures to appointed bodies. These appointed bodies in EU Member States can be Poisons Centres or (other) governmental authorities. In the latter case it is very important that the information is made available to PCs. However, article 45 does not exactly describe what information is required and how it should be notified. At a late stage in the development of the CLP Regulation, under the pressure of all stakeholders (EAPCCT, governmental authorities, industry), this shortcoming was recognised and corrected with paragraph 4. It states that, before January 20, 2012, the EC shall review the possibility to harmonise product notification to the appointed bodies in the EU, including establishing a data exchange format. In 2010 the EC organised two meetings with representatives from the EAPCCT and governmental authorities to discuss the requirements for the notification of product information. The starting document for the discussions was the EAPCCT guideline on product information requirements^8^ from 1989. A new updated version of the guidelines was established and endorsed by the EAPCCT Board in September 2010.

These new EAPCCT guidelines 2010^9^ describe the product information that should be notified by companies in order to facilitate the possibility for PCs to perform an adequate risk assessment after exposure to a product. An important part of the guidelines are the requirements on the composition of a product and the concentration of the ingredients. A full description is required; it is necessary to mention all substances in the mixture (independent of the toxicity) without the use of thresholds. Based on PCs experience, a selection was made for which CLP health hazard classes and categories an exact concentration of substances in products is required (see Table 2). For substances in the remaining health hazards classes and categories, to know the presence in a mixture is more important than to know the actual concentration. For these and all other substances in products, the following defined concentration ranges can be used according to the EAPCCT guidelines: > 0– ≤ 0,1%, >0,1–≤1%, >1–≤3%, >3–≤10%, >10–≤20% >20–≤30%, >30–≤50%, >50–≤75% and > 75%. As PCs may be confronted with very unusual misuse (injection e.g.), a complete accurate composition should be provided on request in exceptional cases.

**Table 2:** EAPCCT guidelines 2010 on the required product composition.^9^ An exact concentration is required for substances with a certain hazard classification according to the CLP Regulation. For all other substances (classified differently or not classified) defined concentration ranges can be used.

CLP Regulation (EC) NO 1272/2008		EAPCCT guidelines 2010	
	
Hazard class	Categories	Exact concentration	Defined concentration ranges
Acute toxicity Oral	1, 2, 3, 4	1, 2, 3	4
Acute toxicity Dermal	1, 2, 3, 4	1, 2, 3	4
Acute toxicity Inhalation	1, 2, 3, 4	1, 2, 3	4
STOT - single exposure	1,2,3	1, 2	3
STOT - repeated exposure	1, 2	1, 2	
Aspiration hazard	1		1
Skin corrosion/irritation	1ABC, 2	1ABC	2
Eye damage/irritation	1, 2	1	2
Respiratory sensitisation	1		1
Skin sensitisation	1		1
Carcinogenicity	1AB, 2		1AB, 2
Germ cell mutagenicity	1AB, 2		1AB, 2
Reproductive toxicity	1AB, 2		1AB, 2
Effects on or via lactation			

STOT: Specific Target Organ Toxicity.

Defined concentration ranges: >0-<0,1%, >0,1-<1%, >1-<3%. <20%, >20-<30%, >30-<50%, >50-<75%, >75%.

The guidelines require information on the identification of the mixture (trade name a.o.), the company submitting the mixture information and the company placing the mixture on the market. Information on the categorisation (describing the intended use), classification, packaging, physical/chemical characteristics and toxicological properties of a mixture is also required.

For companies it would be most convenient if the well-established Safety Data Sheet (SDS) that must be available for hazardous mixtures could be used for product notification. However, not all information as described in the EAPCCT guidelines, especially a precise composition, is available on the SDS. Besides the necessary EAPCCT dataset, the SDS can be provided as additional information for the PC (see below).

In November 2010, the EC organised a workshop where the EAPCCT presented to a broader audience of various Commission Services, competent authorities in the Member States, industry, industry associations and other stakeholders, the requirements on product information necessary for PCs to treat victims of poisonings after exposure to hazardous products. It provided a good basis for further discussion between all stakeholders to harmonise product notification in EU Member States. Stakeholders exchanged their views on the required quality of the product information, especially on the details of the composition that should be provided. Furthermore, the use of a Unique Product Identifier (UPI) to clearly and unambiguously identify a mixture and its specific formula, product categorisation and the data exchange format were discussed. Summaries of the presentations and the complete EAPCCT guidelines 2010 are published by the EC in the Stakeholder workshop report.^9^ After two more meetings on this matter the EC will report (before January 20, 2012) whether harmonisation of product notification can be reached, and how. When possible, the next step will be the development of a legislative proposal to be incorporated into an Annex to the CLP Regulation. This proposal will follow the normal EU legislative procedure with discussions in working groups and a vote by Member States in the relevant regulatory Committee.^9^ If accepted by the EC parliament, the new harmonised notification procedures will be legally implemented in all EU Member States.

### Notification of product information on plant protection products and biocidal products

The requirement to notify product information and the future harmonisation, if achieved, will also apply to plant protection products and biocidal products. The forthcoming Biocidal Products Regulation (expected in 2011) will contain an article on product notification referring to article 45 of the CLP Regulation. The new Plant Protection Products Regulation does not contain such an article on product notification, and thus article 45 on product notification of the CLP Regulation, as a ‘general legislation’ on substances and mixtures, applies directly to this product group.

## REACH Regulation

An important aim of the REACH Regulation, that entered into force on June 1, 2007, is to ensure a high level of protection of human health and the environment from the risks that can be posed by chemicals on the EU market.^10^ Industry is responsible for assessing and managing the risks and to gather all relevant information of substances on these issues. There is an obligation to register this information at the European Chemicals Agency (ECHA) in Helsinki. There are a series of registration deadlines based on the tonnage in which the substance is manufactured or imported and the hazard classification of the substance. After an 11 year phase-in period, all chemicals should be registered by May 31, 2018.

Important for Poisons Centres is that the REACH Regulation will incorporate updated requirements for the Safety Data Sheet in Annex II, amending the Safety Data Sheet Directive. In the discussions on the harmonisation of product notification, this extended and improved SDS is foreseen to be part of the notified product information to PCs and is currently already used for this purpose in several EU Member States.^11^

The REACH Regulation might provide possibilities for further collaboration between PCs and industry.

For the registration, human data is requested from companies and this information is not readily available. The industry could benefit from PCs experience with human exposures to hazardous mixtures and the occurring symptoms.

On the other hand, PCs could benefit from access to the substance information in the ECHA database (at the moment this information is only available for selected governmental authorities), and this possibility should be explored.

### Safety Data Sheet

The SDS is mainly intended to inform professional users on the safe use of the substance or mixture at the workplace. Until now the SDS for mixtures according to the SDS Directive does not provide a detailed product composition, and toxicological information is limited or entirely missing. Improvements in the SDS are expected by the REACH Regulation. For registration of substances, the REACH Regulation requires a technical dossier (containing basic toxicological information), and for some substances, a Chemical Safety Report (CSR) with a ‘human health hazard assessment’ (toxicokinetics, acute effects, irritation/corrosivity a.o.) and exposure scenarios. An exposure scenario includes a.o. information on how the mixture will be used by workers/consumers (duration/frequency) and risk management measures to reduce or avoid direct and indirect exposure. Toxicological information necessary for the registration to ECHA will be incorporated in the SDS and exposure scenarios in an Annex to the SDS.

When the SDS for mixtures will be aligned to the UN-GHS in 2015, the toxicological information will be further extended. This forthcoming new REACH Annex II^12^ specifically states that SDS section 11 is meant for medical professionals and toxicologists. For every health hazard classification, toxicological information should be included. If available, human data should be provided. A further improvement is that if a mixture is not classified for a particular hazard, it should be stated that data are lacking, or data are inconclusive or insufficient for classification. The improved toxicological information in the new SDS is helpful for PCs but an important shortcoming for PC use still remains, because on the SDS only dangerous substances above specified thresholds have to be mentioned. Furthermore, as guidelines on the concentration are not available, wide concentration ranges can be used. The consequence is that adequate risk assessment after exposure is seriously hampered. Additional information on the precise composition remains necessary for PC practise.

Because of the transition from the old to the new classification, at the moment, PCs can be confronted in the SDS ingredients list with a double classification of substances. Suppliers that already classify and label their mixtures according to the new CLP Regulation (an obligation only from June 2015 and voluntary before this date) can use the new REACH Annex II of 1 June 2015 for the compilation of the SDS of the mixture. If a supplier chooses to do so, it is required to provide the classification of the ingredients according to the old Dangerous Substances Directive in addition to the classification according to the new CLP Regulation.

## Cosmetic Products Regulation

Also relevant for Poisons Centres is the development of a Cosmetic Products Notification Portal (CPNP). Article 13 of the new Cosmetic Products Regulation prescribes that companies have to notify, by electronic means, clearly defined product information to the EC. The EC will make the product information electronically available to PCs and governmental authorities. For the implementation of this notification procedure, the EC is currently developing a central database to which cosmetic product information can be uploaded by companies using a secure website. PCs and competent authorities can search, view and download the available product information. Distribution of all product information in an XML-based data exchange format is foreseen to give PCs the opportunity to process the information into their local databases. The CPNP will become operational at the beginning of 2012. From July 11, 2013, the notification of cosmetic products will be a legal obligation.

The EAPCCT and Colipa (the European Cosmetics Association) together with other stakeholders, cooperate with the CPNP development team of the EC in several working groups discussing aspects in the development of frame formulations, categorisation of cosmetic products and IT-related issues.

The working group on frame formulations updates the existing system of frame formulations developed by Colipa in collaboration with the EAPCCT in 2000. This system was used in several EU Member States for either voluntary or obligatory (due to National legislation) notification. The frame formulations are standardised formulations listing the type of ingredients and their maximum concentration in the cosmetic products. Furthermore, based on PCs experience and the hazard classification, cosmetic product ingredients are identified for which an exact concentration should be notified besides the standard frame formulation. Cosmetic products not fitting in a frame formulation and for specified products of high concern (like nail polish removers), the complete qualitative and quantitative formulation needs to be provided.

The working group on categorisation implemented a harmonised categorisation system into the CPNP, a requirement according to article 13 (a) of the Cosmetic Products Regulation. This categorisation system combines the cosmetics part of the German categorisation system^13^ with information from Colipa and industry to cover all cosmetic products.

In the working group on IT-related issues the EAPCCT proposed to implement suitable online search functions, clear screen presentations of product information and versioning of product information enabling PCs to identify formula changes in a cosmetic product. Furthermore, the importance is stressed to align the requirements and notification procedures for cosmetic products with those for the hazardous mixtures. One electronic data exchange format that suits the notification of all (hazardous) mixtures will reduce the costs for the stakeholders involved.^7^

## Conclusion

Poisons Centres in EU Member States have to prepare themselves to handle a substantially increased quantity and quality of information on hazardous mixtures and cosmetic products from 2012 onwards. Besides a change in hazard classification and hazard communication elements for substances and mixtures, the notification of product information by companies will change. For hazardous mixtures the possibility to harmonise the notification in EU Member States is currently reviewed by the EC as laid down in article 45 (paragraph 4) of the CLP Regulation. An important step in this process is the development of the new ‘EAPCCT guidelines 2010’ that describe the information necessary for PCs to adequately perform their task and are the basis for further discussion on product information requirements. It is expected that the REACH Regulation will improve the toxicological information present on the new SDS. The development of a central European database for notification of cosmetic products is already in an advanced stage and the Cosmetic Products Notification Portal (CPNP) will go live early 2012. Details of the data exchange format for both hazardous mixtures and cosmetic products are still under discussion, and where possible, should be aligned.

## Declaration of interest

The authors report no conflict of interest. The authors alone are responsible for the content and writing of the article.
